# Effect of epicatechin consumption on the inflammatory pathway and mitochondria morphology in PBMC from a R350P desminopathy patient: A case report

**DOI:** 10.14814/phy2.16020

**Published:** 2024-04-24

**Authors:** Germán Tapia‐Curimil, Mauricio Castro‐Sepulveda, Hermann Zbinden‐Foncea

**Affiliations:** ^1^ Exercise Physiology and Metabolism Laboratory, School of Kinesiology, Faculty of Medicine Universidad Finis Terrae Santiago Chile; ^2^ Centro de Salud Deportiva Clínica Santa María Santiago Chile; ^3^ Facultad de Ciencias de la Salud Universidad Francisco de Vitoria Madrid España

**Keywords:** desminopathy, epicatechin, inflammation, myopathy, PBMC, TLR4 and mitochondrial dynamics

## Abstract

Desminopathy R350P is a human myopathy that is characterized by the progressive loss of muscle fiber organization. This results in the loss of muscle size, mobility, and strength. In desminopathy, inflammation affects muscle homeostasis and repair, and contributes to progressive muscle deterioration. Mitochondria morphology was also suggested to affect desminopathy progression. Epicatechin (Epi)—a natural compound found in cacao—has been proposed to regulate inflammatory signaling and mitochondria morphology in human and animal models. Hence, we hypothesize chronic Epi consumption to improve inflammatory pathway and mitochondria morphology in the peripheral blood mononuclear cells (PBMCs) of a desminopathy R350P patient. We found that 12 weeks of Epi consumption partially restored TRL4 signaling, indicative of inflammatory signaling and mitochondria morphology in the desminopathy patient. Moreover, Epi consumption improved blood health parameters, including reduced HOMA‐IR and IL‐6 levels in the desminopathy patient. This indicates that Epi consumption could be a useful tool to slow disease progression in desminopathy patients.

## 1 INTRODUCTION

Desmin is a skeletal muscle structural protein that provides stability during muscle contraction. Mutations of the *des* gene can result in aberrant Desmin structures that may lead to desminopathy (DES), a type of myofibrillar myopathy (Kley et al., 2016). Myofibrillar myopathies are progressive skeletal muscle diseases characterized by progressive muscle dysfunction, muscle damage, the presence of protein aggregates, and inflammation. Acute muscle healing involves mounting an immune response that increase inflammation levels (Summan et al., 2006), which in turn contributes to progressive skeletal muscle atrophy (Mukund & Subramaniam, 2020) and affects the muscle's regenerative capacity.

Monocytes are a type of peripheral blood mononuclear cell (PBMC) that regulate the activity and expression of specific receptors that initiate inflammatory signaling pathways (Torres‐Ruiz et al., 2020). The membrane protein Toll‐like receptor 4 (TLR4) triggers an inflammatory response in cells. Upon activation, TLR4 first induces the myeloid differentiation gene 88 (Lu et al., 2008) and then initiates downstream signaling via either the IKK complex that induces IkBα activity, or via the mitogen protein kinases (MAPK) P38, JNK, and ERK. This culminates in NF‐kB activation, interleukin transcription, and the generation of the systemic inflammatory response in cells (Li et al., 2019; Rossol et al., 2011). The myokine IL‐6 is produced in response to inflammation and other stressors, such as skeletal muscle damage (Welc & Clanton, 2013). Inflammatory responses are involved in myofibrillar myopathy repair (Rizzo et al., 2020) and affect mitochondria morphology in human skeletal muscle (Castro‐Sepulveda et al., 2023).

We showed that PBMCs from COVID‐19 patients with acute inflammation present altered mitochondria morphology and mitochondria cristae architecture (Castro‐Sepulveda et al., 2022). Mitochondria are dynamic organelles that play an important role in highly active tissues, including skeletal muscle. Mitochondrial dynamics refers to mitochondria fusion/fission (Castro‐Sepulveda et al., 2023), which is essential for cell viability (Romanello & Sandri, 2015). Human PBMCs are responsive to metabolic states and substrate availability and can be obtained less invasively than muscle biopsies (Castro‐Sepúlveda et al., 2021). PBMCs are thus well suited cell model to investigate the metabolic and inflammatory effects of potential pharmacological tools in desminopathy patients.

Epicatechin (Epi) is a flavonoid found in foods such as cacao. Epi administration affects oxidative stress, inflammatory pathways, mitochondria function, and muscle function in skeletal muscle (Zbinden‐Foncea et al., 2022). Furthermore, Prince et al. (2017) demonstrated that Epi administration reduces TLR4 inflammatory signaling in the skeletal muscle of rats with systemic inflammation. Thus, we hypothesize that chronic Epi consumption by a desminopathy patient would reduce TLR4 inflammatory pathway signaling activity and thereby affect mitochondrial dynamics in the PBMCs.

## 2 METHODS

### 2.1 Case presentation

The DES patient is a 56‐year‐old male with no family history of desminopathy. He was diagnosed in 2006 with desminopathy—R350P mutation of the *des* gene. His two main symptoms are as follows: (a) severe muscular weakness in the lower extremities, the left leg is affected most severely; (b) muscular weakness in the upper left extremity (shoulder and shoulder girdle). The DES patient can only perform some of his basic of daily living activities (eating, sphincter control, and some mobility for writing) independently and is bound to his electric wheelchair. His case has been previously clinically described by Monje et al. (2020). The control subject, the DES patient's 54‐year‐old brother, has no clinical desminopathy symptoms.

### 2.2 Epicatechin administration

The DES subject consumed cocoa powder rich in Epi (100 mg/day) mixed with yoghurt daily for 12 weeks. The control subject consumed just yoghurt for 12 weeks. The Epi content in the cocoa powder was determined by HPLC (634 mg of Epi per 100 g of cocoa powder).

### 2.3 Blood samples

Blood samples were drawn and collected in 10‐mL heparin EDTA tubes and subsequently centrifuged at 4000 rpm for 10 min at 4°C. Plasma was stored at −80°C. The clinical parameters of blood samples were measured by chemiluminescence and dry chemistry in a clinical laboratory (Redlab, Chile).

### 2.4 PBMC isolation

PBMCs were separated under sterile conditions on Ficoll‐Histopaque 1077 (Sigma, Milan, Italy) gradients from ~10 mL of freshly drawn blood (see Castro‐Sepulveda et al., 2022) to obtain ~80% lymphocytes. PBMCs were frozen and stored at −80°C for Western blotting.

### 2.5 Western blotting

PBMCs were homogenized in a lysis buffer containing: 20 mM Tris–HCl (pH 7.5), 1% Triton X‐100, 2 mM EDTA, 20 mM NaF, 1 mM Na2P2O7, 10% glycerol, 150 mM NaCl, 10 mM Na3VO4, 1 mM PMSF, and a protease inhibitor cocktail (Complete TM, Roche Applied Science). Proteins were separated by SDS‐PAGE and transferred to PVDF membranes with a 0.2 μm pore size (1620177, BIO‐RAD). The following antibodies were used: TLR4 (293072, Santa‐Cruz Biotechnology), MyD88 (sc‐74,532, Santa‐Cruz Biotechnology), IKKα (2682S, Cell‐Signaling), IKKβ (8943S, Cell‐Signaling), p‐IKKα/β (Ser176/180) (2697S, Cell‐Signaling), IKBα (4814S, Cell‐Signaling), SAPK/JNK (9252S, Cell‐Signaling), p‐SAPK/JNK (Thr183/Y185) (9251‐S, Cell‐Signaling), p38 MAPK (9212S, Cell‐Signaling), p‐p38 MAPK (Thr180/Y182) (9215S, Cell‐Signaling), p44/42 Erk1/2 MAPK (137F5, Cell‐Signaling), p‐p44/42 Erk1/2 MAPK (Thr202/Tyr204) (197G2, Cell‐Signaling), Optic atrophy 1 (Opa 1, 612,606, BD‐Biosciences), and p‐DRP1 (Ser616) (3455, Cell‐Signaling). Protein expressions were normalized to Ponceau (BM‐1492, Winkler) Red staining and GAPDH (97166S, Cell‐signaling). The secondary antibodies were anti‐rabbit IgG (7074P2, Cell‐signaling) and anti‐Mouse IgG (31430, Invitrogen).

### 2.6 Interleukin analysis

Human IL‐6 and IL‐1Ra were measured by ELISA assays (Abcam commercial kit; IL‐6, ab46042; IL1Ra, ab211650).

### 2.7 Transmission electron microscopy

PBMCs were prepared as previously described (Castro‐Sepúlveda et al., 2021). Sections of 80 nm were cut, mounted on electron microscopy grids, and examined using a transmission electron microscope (Philips, Tecnai 12 at 80 kV). Mitochondria density (%) and size (μm^2^) were assessed in 4–6 cells per subject and 3–10 mitochondria per cell. Mitochondria cristae density (cristae number and length) was assessed as previously described (Castro‐Sepúlveda et al., 2021).

### 2.8 Data analysis

Western blot data are reported as arbitrary units (a.u). Protein content was normalized so that the control subject's value obtained at the PRE measurement was set to 1.0 a.u. The colored area represents the random error, which corresponds to the changes over time (delta) between pre‐ and post‐measurements of the control subject. This analysis was performed based on the high or low response to a stimulus previously reported by Dankel and Loenneke (2020).

## 3 RESULTS

**FIGURE 1:**
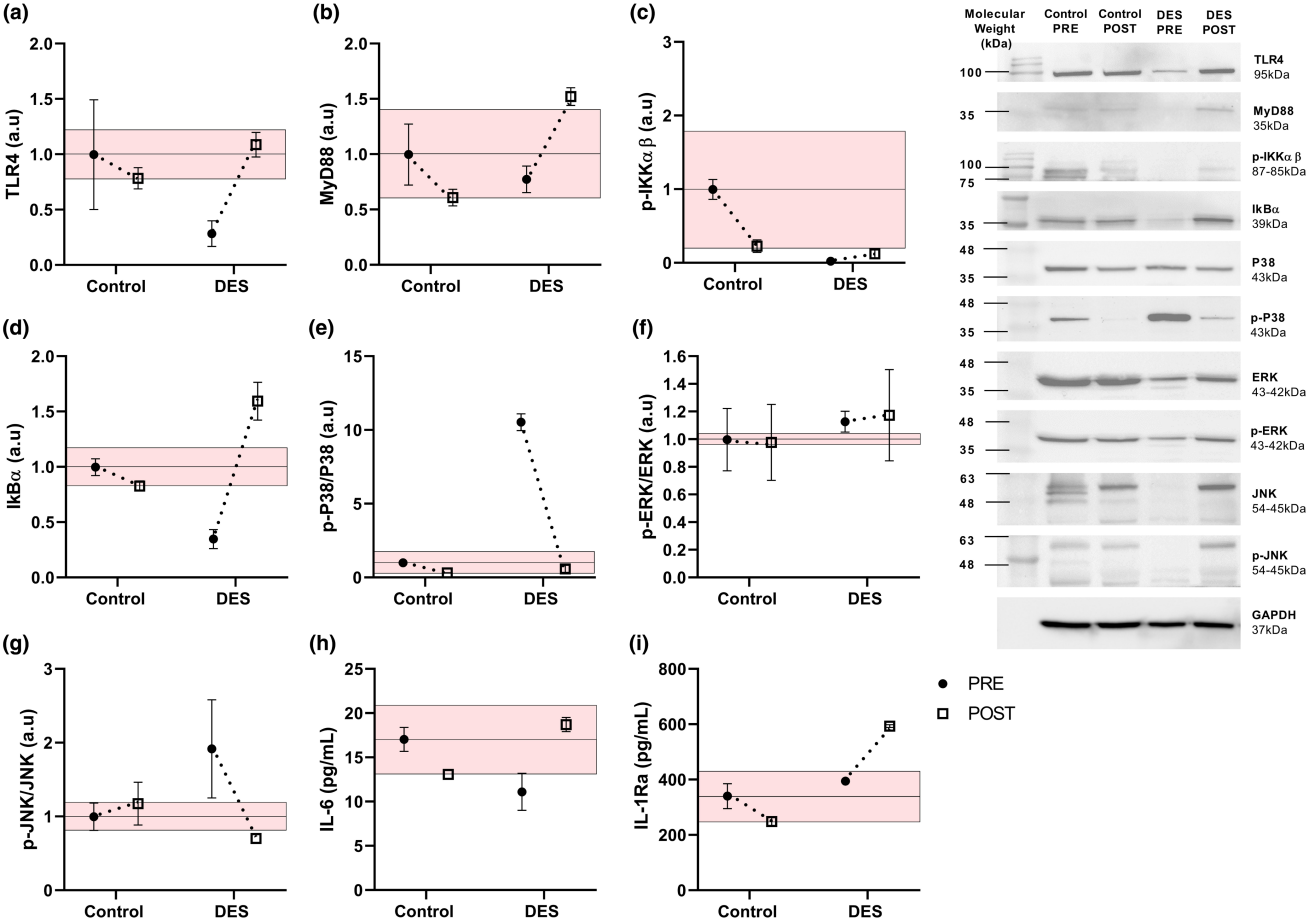
Inflammatory signaling in PBMCs and cytokine levels of the control subject and DES patient. (a) TLR4, (b) MyD88, (c) p‐IKKαβ, (d) IkBα, (e) p‐P38/P38, (f) p‐ERK/ERK, (g) p‐JNK/JNK. Protein levels obtained by Western blot; (h) IL‐6 and (i) IL‐1Ra, plasma concentration obtained by ELISA. The colored area represents the random error.

In the Control, TLR4 decreased from 1.0 a.u PRE to 0.8 a.u POST, while in the DES subject TLR4 increased from 0.3 a.u PRE to 1.1 a.u POST (Figure 1a). MyD88, decreased in the Control from 1.0 a.u PRE to 0.61 a.u POST, but in the DES subject increased from 0.77 a.u PRE to 1.52 a.u POST (Figure 1b).

In the Control p‐IKKαβ decreased from 1.0 a.u PRE to 0.23 a.u POST, while the DES subject increased from 0.02 a.u PRE to 1.12 a.u POST (Figure 1c). IKBα decreased in the Control from 1.0 a.u to 0.83 a.u POST, while the DES subject increased from 0.35 a.u PRE to 1.60 a.u POST (Figure 1d).

In the Control, the p‐P38/P38 ratio decreased from 1.0 a.u PRE to 0.21 a.u POST, while the DES subject showed a more pronounced decrease from 9.32 a.u PRE to 0.48 a.u POST (Figure 1e). The p‐ERK/ERK ratio decreased in the Control from 1.0 PRE to 0.98 POST = 0.98 but increased in the DES subject from 1.13 a.u PRE to 1.18 a.u POST (Figure 1f). The p‐JNK/JNK ratio increased in the Control from 1.0 a.u to 1.18 POST but decreased in the DES subject from 1.92 a.u PRE to 0.70 a.u POST (Figure 1g).

The inflammatory cytokine IL‐6 returned to normal levels in the DES patient after Epi consumption (PRE = 11.1 pg/mL, POST = 18.7 pg/mL) with respect to the Control (PRE = 17.0 pg/mL, POST = 13.1 pg/mL; Figure 1h). Anti‐inflammatory cytokine IL‐1Ra increased in DES patient after Epi consumption (PRE = 394.1 pg/mL, POST = 593.1 pg/mL) with respect to the Control (PRE = 340.1 pg/mL, POST = 248.6 pg/mL; Figure 1i).

**FIGURE 2:**
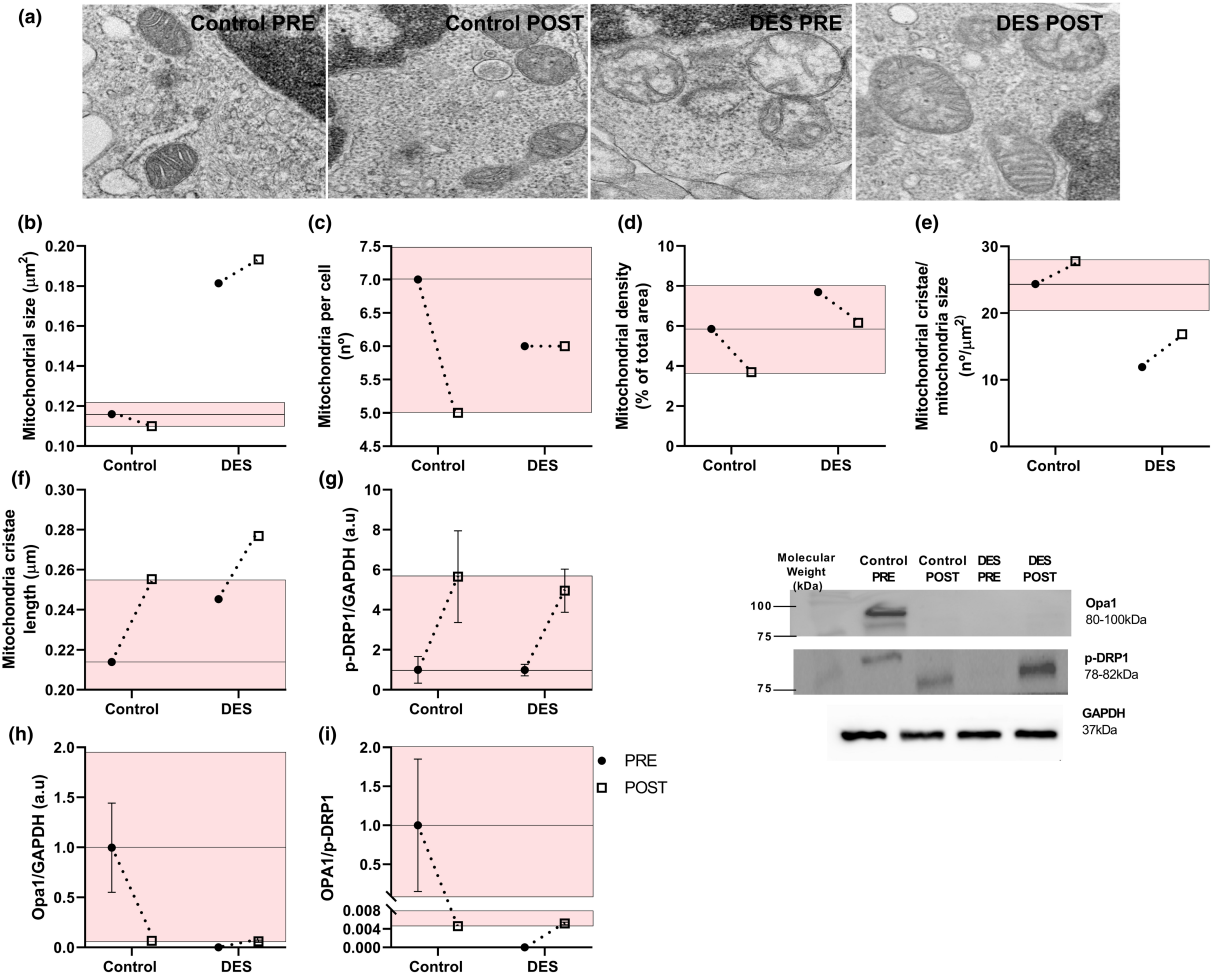
Mitochondrial morphology and mitochondrial dynamic protein levels. (a) Representative image of mitochondria from PBMCs of the DES patient and the control subject, (b) mitochondrial size, (c) cellular mitochondrial number, and (d) mitochondrial density, (e) mitochondrial cristae relative to mitochondrial size, (f) mitochondrial cristae length, (g) p‐DRP1, (h) OPA1, and (i) ratio OPA1/p‐DRP1. The colored area represents the random error by change ratio of control between PRE and POST.

Mitochondria remained larger in the DES subject after Epi consumption (PRE = 0.18 μm, POST = 0.19 μm), with respect to the Control (PRE = 0.11 μm, POST = 0.11 μm; Figure 2b). Cellular mitochondria number was unaffected in the DES subject (PRE = 6, POST = 6), but reduced in the control subject (PRE = 7, POST = 5; Figure 2c). Mitochondria density per total area remains in the random error range in DES (PRE = 7.69%, POST = 6.16%) and control (PRE = 5.85%, POST = 3.69%; Figure 2d). Mitochondria cristae number relative to mitochondria size increased in DES but (PRE = 11.9 n/μm, POST = 16.8 n/μm) did not reach control levels (PRE = 24.4 n/μm, POST = 27.7 n/μm; Figure 2e). Mitochondria cristae grew larger in DES (PRE = 0.24 μm, POST = 0.27 μm) than the control (PRE = 0.21 μm, POST = 0.25 μm; Figure 2f).

Mitochondrial dynamics was evaluated by determining p‐DRP1 and Opa1 levels. p‐DRP1 levels increased in both the DES (PRE = 0.99 a.u, POST = 4.95 a.u) and the Control subject (PRE = 1.0 a.u, POST = 5.66 a.u; Figure 2g). OPA1 levels decreased in the control subject (PRE = 1.0 a.u, POST = 0.068 a.u), and the DES subject (PRE = 0.0 a.u, POST = 0.064 a.u; Figure 2h). In the control subject the OPA1/p‐DRP1 ratio decreased (PRE = 1.0 a.u, POST = 0.004 a.u), whereas in the DES subject it increased to the normal values (PRE = 0.0 a.u, POST = 0.005 a.u; Figure 2i).

C‐reactive protein (CRP) levels increased in the DES patient from 8.6 mg/dL PRE to 12.7 mg/dL POST, but remained constant in the control (6 mg/dL PRE and 6.3 mg/dL POST). Creatinine levels were unchanged in control and DES. HOMA‐IR scores decreased in the DES patient from 10.84 PRE to 8.88 POST, as in the Control subject from 2.0 PRE and 1.71 POST (Table 1).

**TABLE 1:** Clinical parameters of blood samples.

	Control			DES			Clinical value	
	PRE	POST	Change	PRE	POST	Change	Lower	Top
CRP (mg/L)	6	6.3	0.3	8.6	12.7	4.1	<10	
Creatinine (mg/dL)	0.85	0.8	−0.05	0.25	0.2	−0.05	0.66	1.25
Insulin (uUI/ml)	7.3	6.54	−0.76	41.43	28.56	−12.87	2.6	24.9
Glycemia (mg/dL)	111	106	−5	106	126	20	75	110
HOMA‐IR	2.00	1.71	−0.28	10.84	8.88	−1.95		
Uric nitrogen (mg/dL)	14	10	−4	22	14	−8	9	20
Uric acid (mg/dL)	4.8	5.5	0.7	6	7.3	1.3	3.5	8.5
Total bilirubin (mg/dl)	0.599	0.36	−0.239	0.34	0.42	0.08	0.2	1.3
GOT (U/L)	<13	19		16	16	0	17	59
Lactate dehydrogenase (U/L)	200	160	−40	168	155	−13	120	246
Phosphorous (mg/dL)	2.8	2.6	−0.2	2.8	3.6	0.8	2.5	4.5
Total proteins (g/dL)	7.6	7.2	−0.4	6.9	6.9	0	6.3	8.2
Albumin (g/dL)	>5.1	4.8		4.4	4.3	−0.1	3.5	5
Total cholesterol (mg/dL)	179	157	−22	160	164	4		
Col HDL (mg/dL)	54	49	−5	42	38	−4	41	59
Triglycerides (mg/dL)	119	96	−23	183	174	−9		
Cholesterol VLDL (mg/dL)	23.8	19.2	−4.6	36.6	34.8	−1.8	0	40
Cholesterol LDL (mg/dL)	101	88.8	−12.2	81.4	91.2	9.8	0	150
Cholesterol /HDL	3.3	3.2	−0.1	3.8	4.3	0.5		

## 4 DISCUSSION

Our results indicate that 12 weeks of Epi consumption restored the level of certain inflammatory pathway proteins, such as TLR4 and p‐P38/P38, in the DES patient's PBMCs. Similarly, IL‐6 returned to normal levels, and the anti‐inflammatory cytokine IL‐1Ra levels became even higher in the DES patient than in the control. After the Epi intervention, the DES patient increased mitochondria cristae length and fusion/fission mitochondrial dynamic ratio proteins. Creatinine was unaffected in the DES patient. Furthermore, Epi consumption decreased HOMA‐IR in the DES patient.

Inflammation is a crucial response in myopathies to promote the growth and repair of skeletal muscle (Mukund & Subramaniam, 2020). In mouse models of Duchenne muscular dystrophy, TRL4 was identified to be critical in muscle tissue repair (Petrof, 2022). In light sepsis in humans, an acute inflammatory response inducing the TLR4 pathway and inflammatory cytokine production is beneficial, but in severe sepsis these components are dysregulated, preventing inflammatory cytokine production by the PBMCs (Brunialti et al., 2006). Our study provides the first insights of Epi's effects on inflammatory signaling and cytokine production in a desminopathy patient's PMBCs, namely that certain inflammatory signal proteins return to their normal levels.

Accordingly, the grape polyphenol Malvidin diminished the inflammatory response in human PBMCs following LPS in vitro stimulation (Bastin et al., 2020). Barrera‐Reyes et al. (2019) showed that healthy young adults consuming Epi acutely from cocoa modulated their PBMC's inflammatory response. Our data indicates the DES patient's IL‐6 levels rose after Epi administration, which concurs with his increased TLR4 levels. This suggests that polyphenols, specifically Epi, may increase the protein levels of TLR4 pathway components in PBMCs, which is necessary to initiate tissue repair (Cooke, 2019).

Fix et al. (2019) showed that chronically elevated IL‐6 levels in C2C12 myotubes induces DRP‐1 and FIS‐1 expression, which are both involved in mitochondria protein dynamics. Similarly to Navarrete‐Yañez et al. (2020) who showed Epi to increase plasma IL‐1ra in mice with inflammation, our data indicates that IL‐1ra increased after Epi consumption in the DES subject compared to Control. Similarly, Bartsakoulia et al. (2018) found an imbalance in mitochondria fission/fusion dynamics, and showed aberrant mitochondria cristae in fibroblasts from a human myopathy patient. Interestingly, our DES patient has aberrant PBMC mitochondria cristae prior to, but increased cristae length following Epi consumption. This suggests that Epi affects mitochondria fusion/fission dynamics, morphology, and mitochondria cristae density in the desminopathy patient PMBCs.

A potential limitation is that we did not have a positive control or plasma marker to indicate Epi absorption. However, Actis‐Goretta et al. (2013) showed that almost 46% of Epi was absorbed, so we speculate that a similar percentage was absorbed during the 12 weeks of Epi supplementation.

In conclusion, our results indicate that Epi consumption could regulate inflammatory signaling in desminopathy patients, and restore it to normal levels. This supports the idea that Epi affects inflammation to control systemic homeostasis of the immune response and be a potential trigger for repair. A future line of research would be to see if similar results can be obtained in the skeletal muscle of patients with desminopathy after Epi consumption, considering that these results correspond to a single patient.

## FUNDING INFORMATION

This research was supported by the Funds International of Research from the University Finis Terrae (HZ‐F) and a generous gift from the Bertin‐Barbe Family.

## ETHICS STATEMENT

The participants provided a written informed consent which was approved by the Finis Terrae University's scientific ethics committee.
